# Rare but Still There: An Interesting Case of Cytokeratin 20-Negative Merkel Cell Carcinoma

**DOI:** 10.7759/cureus.55612

**Published:** 2024-03-05

**Authors:** Amna Zahid, Arsalan Sheikh

**Affiliations:** 1 Dermatology, Ormskirk District General Hospital (Mersey and West Lancashire Teaching Hospitals), Ormskirk, GBR

**Keywords:** cutaneous, neuroendocrine, interesting, merkel cell carcinoma, cytokeratin 20 negative

## Abstract

Merkel cell carcinoma (MCC) of the skin is a rare and aggressive primary neuroendocrine tumor that mainly involves sun-exposed areas and can metastasize to other parts of the body. Due to varied clinical features and the sharing of similar histological features with other neuroendocrine tumors, diagnosis can be challenging. Therefore, immunohistochemistry plays an important role in diagnosis, and the characteristic perinuclear staining with cytokeratin 20 (CK 20) helps to differentiate it from other morphologically similar tumors, especially metastatic small cell carcinoma of the lung. We describe an interesting case of a 78-year-old female who was referred by a general practitioner (GP) with a few months’ history of asymptomatic, rapidly enlarging, erythematous, nodular lesion on her left upper arm. Due to clinical findings and the location of the lesion on the sun-exposed area, wide differential diagnoses were considered. The lesion was excised for histological diagnosis. Surprisingly, morphological features favour the diagnosis of a neuroendocrine tumor. However, histological features including immunohistochemistry rendered it difficult to differentiate between primary cutaneous neuroendocrine carcinoma (Merkel cell CA) and metastatic small cell carcinoma of the lung due to the lack of specific and sensitive marker of CK 20 on immunohistochemistry. Subsequently, the patient had computer tomography of the chest/abdomen and pelvis (CTTAP) and positron emission tomography (PET) scans to rule out underlying primary malignancy. The case was also discussed at local and specialist skin multidisciplinary team meetings (MDT) including neuroendocrine MDT and a consensus diagnosis of Merkel cell carcinoma of the skin with negative CK 20 was established.

## Introduction

Merkel cell carcinoma (MCC) is a rare, aggressive skin cancer with neuroendocrine features and mainly involves sun-exposed body areas. The incidence of MCC varies in different regions of the world; however, in the Caucasian population, it is 0.23 per 100,000. It mainly affects older people and has a male predominance. Major risk factors include advancing age, UV light exposure, and immunosuppression. Tumor pathogenesis is mainly linked to UV radiation-induced DNA damage and/or Merkel cell polyomavirus (MCPyV). Merkel cell polyomavirus's association with the pathogenesis of MCC was discovered in 2008, and it transformed the understanding of the oncogenesis process involved in the development of MCC in MCPyV-positive patients [1,2]. 

On immunohistochemistry, 95 percent of the cases express cytokeratin 20 (CK 20) [3]. A negative CK 20 staining makes the diagnosis of MCC more challenging, and it is mostly related to an underlying small cell lung cancer [4,5]. MCC presents as a rapidly enlarging, single nodule that clinically could mimic basal cell carcinoma and is usually asymptomatic. It can be localized but also carries a high potential risk of metastasis. Depending upon the extent of the cancer, the treatment varies from surgical excision to radiotherapy and/or systemic immunotherapy/chemotherapy.

## Case presentation

**Figure 1:**
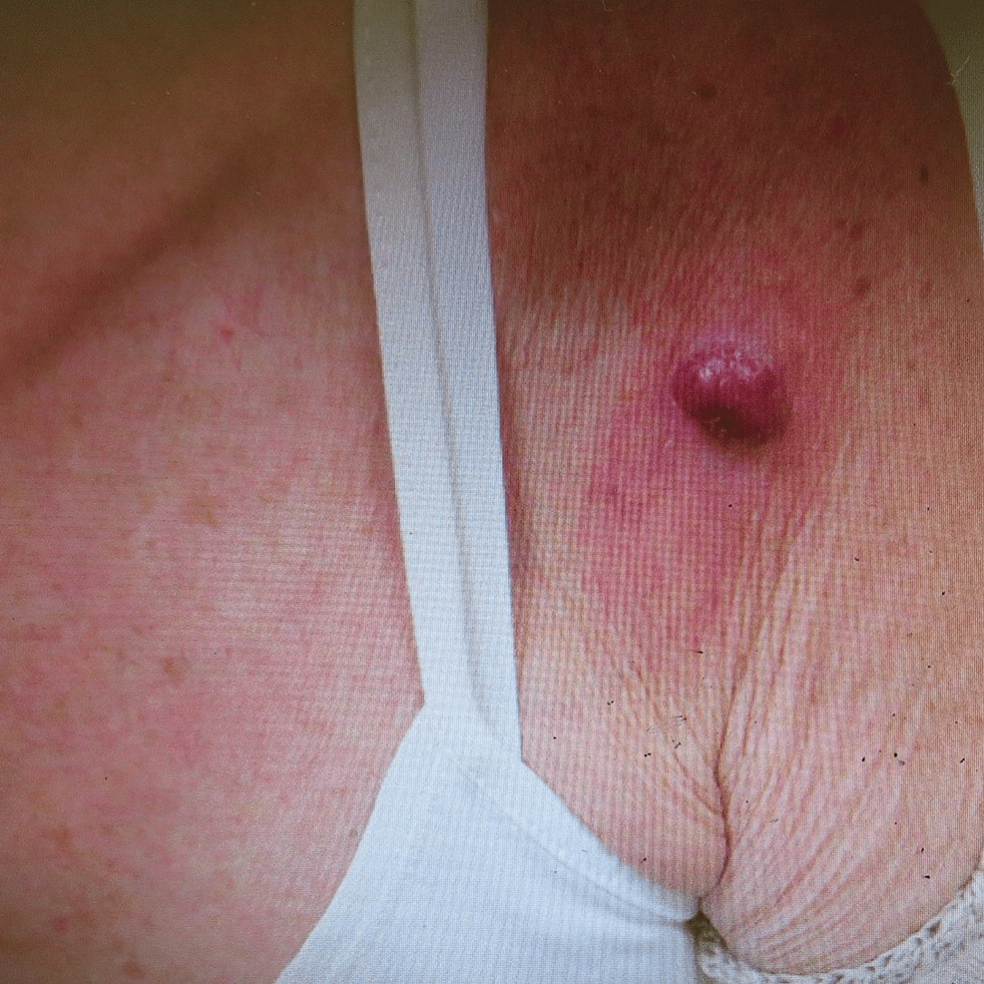
Erythematous nodular lesion showing mild scaling on the surface.

This 78-year-old female was referred to us under a suspected skin cancer pathway by her GP with a lesion on her left upper arm which had been present for a few months and was increasing in size. It became redder relatively recently, otherwise it was asymptomatic. There was no personal or family history of skin cancers; however, she had Fitzpatrick type 2 skin with a previous history of high sun exposure with sunbathing. She was a lifelong nonsmoker and never worked outdoors. There was no history of immunosuppression. On examination, there was an approximately 15 mm erythematous, raised nodular lesion on the left upper arm with a slight irregular border and mild scaling on the surface (Figure 1).

**Figure 2:**
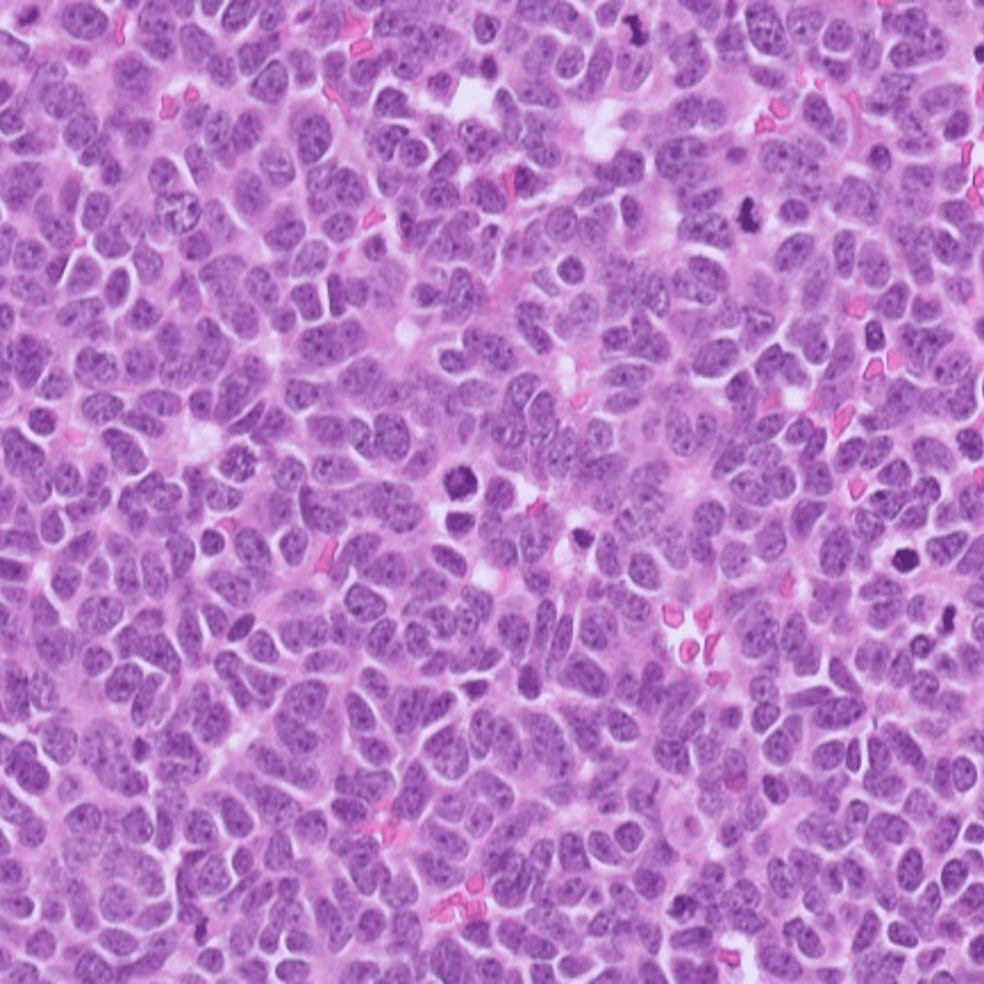
Nests of basophilic cells with fine chromatin and multiple mitoses (hematoxylin and eosin, 100x).

Clinical differential diagnoses included amelanotic melanoma, basal cell carcinoma (BCC), and squamous cell carcinoma. The lesion was excised and sent for histology which revealed a poorly differentiated neuroendocrine tumor. Histology demonstrated nodules of basophilic cells showing fine chromatin and conspicuous mitoses (Figure 2).

**Figure 3:**
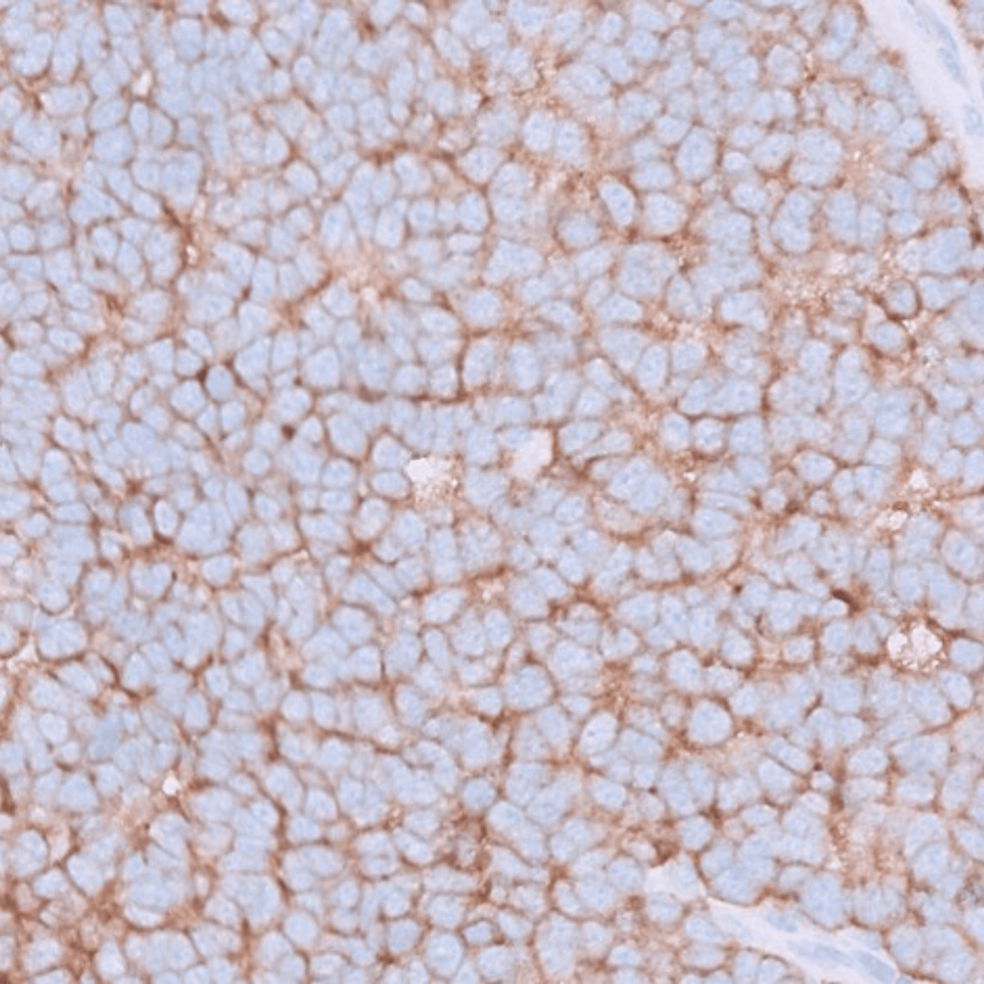
Positive synaptophysin (an immunostain) staining, which highlights the neuroendocrine origin of the cells.

Immunohistochemistry staining was negative for cytokeratin 20, which is a sensitive and specific marker for Merkel cell carcinoma and differentiates it reliably from the cutaneous manifestation of small cell lung carcinoma. However, staining was positive for cluster of differentiation 56 (CD56), synaptophysin confirming the neuroendocrine origin of cells (Figure 3). CD34, Melanin-A, S-100, protein 63 (p63), and CK5/6 markers were negative, thus ruling out lymphoma, melanoma, and other cutaneous malignancies.

The case was discussed at the local skin multidisciplinary team (MDT), specialist skin MDT, and neuro-endocrine MDT due to the diagnostic challenge it posed given the skin lesion and negative CK 20. Initially, there was a suspicion of a secondary tumor arising from small cell carcinoma of the lung due to negative immunohistochemistry of the CK 20 marker. To evaluate this, CTTAP was performed which ruled out any underlying malignancy. The outcome of the neuroendocrine MDT was to perform a PET scan, which further excluded malignancy other than the concerned skin lesion. After discussions at MDTs and reviewing results, a diagnosis of Merkel cell carcinoma with negative CK 20 was made. The patient subsequently had a wide local excision and sentinel lymph node biopsy which yielded no lymph node involvement. She has been kept under skin cancer follow-up for regular reviews and scans for 5 years, and there has been no recurrence or metastases so far.

## Discussion

Merkel cell carcinoma is a rare aggressive cutaneous neuroendocrine tumor that has a propensity for local and distant metastases and has a high recurrence rate. The tumor originates from Merkel cells that are present in the basal layer of the epidermis near nerve endings and are involved in soft touch stimulation. They express many immunohistochemical markers, such as chromogranin A, synaptophysin, and CK 20. It has an estimated incidence of 0.23 per 100,000 people in Caucasian populations. It mainly affects people over the age of 50 years and is slightly more common in men than women. The major risk factors include high UV exposure and conditions that can suppress immunity, such as cancers, HIV, and certain drugs [6]. Recently, studies have shown that human polyomavirus is associated with the pathogenesis of Merkel cell carcinoma, which includes integration of the polyomavirus genome and Merkel cell genetic material can lead to mutations and hence the origin of carcinoma [7,8].

It presents as a rapidly enlarging painless red nodule which occurs mainly on sun-exposed body parts, especially head and neck region and upper extremities. Spread is mainly through the lymphatic system and can metastasize to lymph nodes of the neck, axillae, and groin [9]. Differential diagnoses include BCC, dermatofibroma, lymphoma, or amelanotic melanoma. Diagnosis requires a biopsy of the lesion and immunohistochemistry helps to differentiate it from other cutaneous malignancies and paraneoplastic skin manifestations of small cell carcinoma of the lung [10,11].

Histology shows densely blue cells mainly present in the dermis with the involvement of overlying epidermis and subcutaneous tissue in some cases. Tumor cells are organized in the form of sheets, nests, or trabeculae. Mitosis is quite conspicuous and lymphovascular invasion is common [3]. CK 20 is a type 1 cytokeratin, which is a major cellular protein present in normal cells of the stomach, intestine, urothelium, and in Merkel cells in the skin. Positive tumor cells characteristically express cytokeratin as a dot-like pattern around the nucleus in the form of globules when stained with antibodies directed to CK 20. In immunohistochemistry, the expression of CK 20 is a sensitive and specific marker for Merkel cell carcinoma as it helps in distinguishing it from small cell lung carcinoma as both share morphological similarities [12-14]. However, it has been established that 5% of the MCCs are CK 20 negative and one-third of small cell lung carcinomas can be positive for CK 20. This implies to our case in discussion where tumor cells were negative for CK 20 staining, but further investigations, including CT and PET scanning, were negative for any primaries. The final diagnosis of Merkel cell carcinoma was the diagnosis of exclusion after history, examination, examining characteristic histological features, and being unable to find the underlying metastatic primary.

Following histological confirmation, a biopsy of the sentinel lymph node is considered, and if it is positive then full body imaging is required to detect distant metastases like CT scan or PET scanning [15]. The standard treatment of the tumor localized to the skin is the wide local excision. Regional nodal involvement needs surgical dissection and/or radiotherapy. For distant metastases, radiotherapy and/or systemic chemotherapy may improve quality of life [16]. Overall, the 5-year survival rate is approximately 50% [17]. Worse prognostic factors include advancing age, immunosuppression, multiple comorbidities, and the presence of metastases at the time of diagnosis [18].

## Conclusions

MCC is a rare and aggressive cutaneous malignancy of neuroendocrine origin with a 5-year overall survival rate of approximately 50%. Due to non-specific clinical features and a morphological analogy between Merkel cell cancer and the cutaneous manifestation of small cell lung cancer, diagnosis can be challenging. Immunohistochemistry staining with CK 20 plays an important role in distinguishing the two entities, which is positive in 95% of cases of MCC. The absence of staining renders it extremely difficult to differentiate MCC from other morphologically similar tumors and requires a more comprehensive work-up, as in our case, and careful, informed decision-making.
